# The Buffalo Medical and Surgical Journal

**Published:** 1880-08

**Authors:** 


### Editorial.

---

### THE BUFFALO MEDICAL AND SURGICAL JOURNAL. —THE TWENTIETH VOLUME.

The twentieth volume of the Journal, the second under the present editorial management, opens with this number. One year ago, in undertaking the duties and responsibilities of medical journalism, assurances were given of earnest effort and assiduous labor in the untried field upon which at that time we were entering. How far the promises then made, and the designs and objects unfolded in our salutatory, have been fulfilled, we modestly leave to the public, especially to those who have generously tendered their support and assistance, to decide. A largely increased circulation, with the most cordial endorsement of the position we have taken upon all the leading questions, affecting the vital interests of the medical profession, are among the gratifying evidences of approval of our efforts.

In commencing a new volume, we feel from our brief experience, that the profession will lend a liberal support to this, as well as to every honest effort to advance its interests, to elevate its tone, and to promote its laudable objects. We desire to reiterate the sentiments heretofore expressed in our pages, that the Journal in the future will occupy no uncertain or equivocal positions on questions which concern the great interests of the medical profession, nor will we fail to improve opportunities, as they may arise, to discuss educational and ethical questions, in the most independent manner, or to advocate principles with a view to correct errors into which the profession may have drifted in the past.

We are proud to recognize in the American profession, elements and germs of the fairest promise for the future of medical science. The energy and enterprise required of our people to develop the material resources of a new country, have brought out strong national characteristics, extending to every industry, mechanical and commercial, and permeating the social and intellectual tastes and habits of our people. It would be a matter of surprise if the medical profession in its growth were uninfluenced by the nature of the soil or the peculiarities of climate, or failed to breathe in the contagion of free and independent thought, peculiar to our American civilization. As a body, we are rapidly emerging from this intensely practical period in our professional history. A wider culture and habits of closer study and research are among the propitious omens observed at the present time. Our medical literature already commands favorable recognition both at home and abroad. The Medical Press may justly be considered an important factor among the agencies in operation for the advancement of medical science. In this department we shall continue to devote our best energies, hoping to contribute something to the storehouse of human knowledge, and through the medium of the JOURNAL to disseminate through our widely scattered constituency, in a concise and well-digested form, valuable data to aid the profession in its beneficent work for humanity.

